# ADHD in Intellectual Disability Audit: Diagnosis, Medication and Monitoring

**DOI:** 10.1192/bjo.2025.10675

**Published:** 2025-06-20

**Authors:** Shoyoye Oluwadamilola, Iqbal Mehak, Bennett Sharna, Hemmings Colin, Jordanova Vesna

**Affiliations:** Kent and Medway NHS and Social Care Partnership Trust, Kent, United Kingdom

## Abstract

**Aims:** Attention Deficit Hyperactivity Disorder (ADHD) is more prevalent in adults with an intellectual disability (ID). NHS Digital reported the prevalence of ADHD in the ID population to be 9% in 2023–2024, compared with 1.2% in those without ID. Understanding the prevalence of ADHD within our Mental Health of Learning Disabilities (MHLD) team is crucial for tailoring our services accordingly and improving patient care. We decided to complete an audit across Kent and Medway to assess the degree of service demand arising from the diagnosis and treatment of ADHD in patients already open to the MHLD service, and to assess our adherence to NICE guidance for medication monitoring for ADHD medications.

**Methods:** A cross-sectional study of all patients currently open to MHLD was conducted. Total numbers across MHLD were recorded, as well as the split between the East and West Kent caseloads. All case notes, clinic letters and GP records were reviewed to identify whether a diagnosis of ADHD (or possible ADHD) was present. Once identified, a deep dive of patient records took place to check medication history and GP monitoring. Information was collated about the type of medication prescribed and length of prescription and whether monitoring had been carried out over the past 6 months in accordance with NICE guidance.

**Results:** We found that 15% (N=97) of all MHLD patients (N=629) had a confirmed diagnosis of ADHD, with 65.5% male and 34.5% female. The mean age of these patients was 24.6. Of those with confirmed diagnoses of ADHD, 43% (N=42) were prescribed medication. The most commonly prescribed medication was methylphenidate (62%), followed by atomoxetine (14%) and lisdexamfetamine (9.5%). Most patients had been on ADHD medication for less than 1 year (31%), with only 7% of patients being prescribed ADHD medication for over 10 years. With regards to medication monitoring, for those prescribed ADHD medication, 83% had their weight measured in the last 6 months, 76.5% had their pulse measured and 64% had their blood pressure measured.

**Conclusion:** This audit suggests that the rate of ID and ADHD in our clinical sample is higher than the estimated population prevalence. This will have implications for service development and training requirements, meaning that clear pathways will need to be established, with available resources and adequate monitoring in place to ensure the needs of our patient group are being met, and current NICE guidance is adhered to.

